# Transition from driving to driving-cessation: experience of older persons and caregivers: a descriptive qualitative design

**DOI:** 10.1186/s12877-024-04835-3

**Published:** 2024-03-04

**Authors:** Camille Savoie, Philippe Voyer, Martin Lavallière, Suzanne Bouchard

**Affiliations:** 1 Faculty of Nursing Science, Laval University, 1050 Rue de la Médecine, G1V 0A6 Québec (Québec), Canada; 2 Department of Health Sciences, Université du Québec à Chicoutimi, 555 boulevard de l’Université, G7H 2B1 Chicoutimi (Québec), Canada

**Keywords:** Older driver, Driving-cessation, Older persons, Caregivers, Qualitative

## Abstract

**Background:**

For some older persons, driving is essential to maintain their daily activities and engagement with society. Unfortunately, some will have to stop driving, as they age. Driving-cessation is an important transition for older persons and caregivers, well known to cause significant challenges and consequences. This study aimed to describe the experience of older persons and caregivers in the transition from driving to ceasing to drive.

**Methods:**

Within a descriptive qualitative design, semi-structured interviews were undertaken with older persons (*n* = 8) and caregivers (*n* = 6) from the city of Québec (Quebec, Canada), from November 2020 to March 2021. Using an inductive approach, the qualitative data was analyzed with the content analysis method.

**Results:**

Some older persons had never thought they might someday lose their driver’s license. The process of legislative assessment was unknown by almost all older persons and caregivers. The process was therefore very stressful for the research participants. Driving-cessation is a difficult transition that is associated with loss of independence, freedom, spontaneity, and autonomy. Qualitative analysis of data showed different factors that positively or negatively influence the experience of ceasing to drive, such as the older person’s ownership of the decision, the presence of a network of friends and family, and self-criticism. There was significant impact related to driving-cessation for caregivers, such as assuming the entire burden of travel, psychologically supporting older persons in their grief, and navigating the driver’s licensing system.

**Conclusions:**

These study results could help organizations and healthcare professionals to better accompany and support older drivers and caregivers in the transition from driving to driving-cessation.

**Trial registration:**

None.

## Background

Driving is central to the lives of many older adults. In fact, it is the primary mode of transportation for older people in Canada, United States, and in many other countries [1–3]. As the population ages, there has been an increase in the number of licensed drivers aged 65 and over, in recent decades [4]. Driving is a complex domestic life activity, requiring numerous cognitive, perceptual, visual and psychomotor demands, and takes place in a dynamic environment [5]. It is recognized as an important determinant of the older persons’ autonomy and independence [6].

Hanging up one’s keys is an important transition in the lives of older adults that involves many challenges [7–10]. Indeed, this transition is accompanied by practical needs, such as the search for alternative means of transportation, informational needs, but also emotional needs, such as support in coping with the grief of losing one’s driver’s license [11–13]. Considering the strong symbolism attached to the automobile, in certain cases, the acceptance of the decision to stop driving is not self-evident for older persons or their loved ones [14, 15]. For this age group in particular, giving up driving is associated with numerous negative collateral effects, such as loss of autonomy, mobility and freedom [16, 17]. Studies report an increase in depressive symptoms [13, 18, 19] and an accelerated deterioration in general health [20]. Approximately 20% of older persons who lose their driver’s license experience depression afterwards [19], several researchers have even gone so far as to suggest that giving up driving doubles an older person’s risk of subsequent depression [18]. The individual’s lifestyle is also affected, with fewer activities outside the home [21] and increased social isolation [13, 18, 22, 23]. The impact of losing a driver’s license affects not only the older person, but also those close to him/her. Older persons suddenly become dependent on those around them for their transportation needs, and this can sometimes create tensions [10], become a burden for caregivers [24] and create changes in family dynamics [8]. Caregivers are often at a loss when it comes to the help they should provide to older persons. They expressed the need for consistent and relevant support and information to better support seniors in the grieving process.

Withdrawal of a driver’s license and the needs that go with it have received little attention in the literature until now. However, Pickard et al. [12] argued that older people who have stopped or will stop driving need to be adequately informed and supported at this important stage in their lives. The literature highlights the importance of talking about the possibility of losing one’s driver’s license, early in the aging process [25, 26]. MacDonald and Hebert [27] deplore the lack of a program to prepare older adults and their caregivers for this transition. It is therefore essential to gain a better understanding of the experience of older persons and their loved ones, to provide them with adequate support and meet their needs in the transition from driving to definitive driving-cessation. However, few studies have focused on the experience of older persons and caregivers in the transition from driver to passenger, particularly in Canada. Finally, there are no studies in the literature on this subject in Quebec. The aim of this research was therefore to describe the experience of older persons and caregivers in the transition from driving to ceasing to drive. More specifically, this research aimed to answer the following research questions:

1. How do older persons describe their needs during the transition from driving to not driving?
2. What is the experience of caregivers accompanying older persons in their transition from driving to ceasing to drive?

## Methods

### Study design

A descriptive qualitative design was chosen to meet the research objective and questions [33]. This type of design highlights the perspective of those who live the health experience, with a view to developing interventions in the future [34]. According to Sandelowski (2000) [35], such an approach is appropriate to briefly describe a little-known phenomenon.

This study is part of an ongoing larger research project aimed to develop and evaluate the acceptability and feasibility of a road safety assessment and support guide for older persons, intended for the nursing profession.

### Theoretical framework

Afaf Ibrahim Meleis’s intermediate theory of transition [36] was chosen as the theoretical basis for this study. The theory comprises four central concepts: nature of transition, transition conditions, patterns of response and nursing therapeutics. The use of this theory in this research promotes understanding of the transition experienced by older people and caregivers. In addition, this theory can be used to develop and evaluate interventions aimed at facilitating transition and promoting well-being and mastery of the changes associated with a successful transition [36]. The theory guided the development of research questions, interview guides and data analysis.

### Setting and participants

A total of eight older persons and six caregivers were recruited, using convenience sampling so that participants are selected on the basis of precise criteria to make the best possible contribution to the purpose of the study [33]. Older persons and caregivers were not related and were recruited separately. Older persons were recruited from a variety of locations in the community from the city of Québec (Quebec, Canada) (private older persons’ residences, community organizations) by displaying promotional posters of the study. Older persons interested in participating in the study could contact the first author via the contact details on the poster. When the older persons called, the first author did the recruiting, making sure they met the inclusion criteria, explaining the project and answering any questions. The call was also used to schedule the interview. Older adults had to meet the following inclusion criteria: Be 65 years old or older, speak French, be a registered patient in a family medicine group, hold a valid class 5 driver’s license (passenger vehicles) or have had their driver’s license revoked, within the last year. On the other hand, older persons could not participate in the study if they had a diagnosis of mild or major neurocognitive disorder or had cognitive deficits with a functional impact on one or more activities of daily living (feeding, dressing, hygiene care, mobilization, and elimination). Caregivers were recruited from caregivers’ associations, private older persons’ residences and private occupational therapy clinics of the city of Québec (Quebec, Canada). Promotional posters were also distributed to these associations and locations. Recruitment was carried out in the same way as with the older persons. Caregivers interested in participating in the study could contact the first author via the contact details on the poster. When the caregivers called, the first author did the recruiting, making sure they met the inclusion criteria, explaining the project and answering any questions. To take part in the study, caregivers had to speak French and have accompanied an older adult through the process of assessment and driving cessation, within the last two years. Relatives with cognitive deficits or a diagnosis of mild or major neurocognitive disorder were excluded. To rule out the presence of cognitive deficits, participants were questioned directly. Moreover, the first author, which conduct the interviews, has advanced clinical expertise in geriatrics, which enabled her to screen the presence of cognitive deficits in participants’ speech.

### Ethics

The study was conducted following the Declaration of Helsinki, was approved by the CIUSSS de la Capitale-Nationale Ethics Board (#2021 − 1871). All participants were briefed on the requirements of the study and invited to read and sign an informed consent declaration. An informed consent was obtained from all participants. Since the interviews were conducted by telephone, the consent form was sent to the participants prior to the interview. At the start of the interview, a verbal consent script was read to the participant, and their verbal consent was recorded if they agreed to participate. To preserve anonymity, participants were coded and only the first three authors had access to the codes.

### Data collection

Following public health recommendations in the context of the COVID-19 pandemic, all interviews were conducted in French by telephone by the first author, from November 2020 to March 2021. Semi-structured interviews lasting approximately 45 min were conducted with the recruited older adults, using interview guide. The purpose of the interviews was to gather data on the older persons’ experiences of the clinical and/or driving assessment and cessation process. Here are some sample questions: How did you feel immediately after you stopped driving? What effect did stopping driving have on your life? This experience data enabled us to better understand the needs underlying this process and, ultimately, to better support the older persons.

Semi-structured interviews lasting approximately 45 min were also conducted with recruited family members, using a second interview guide. Here are some sample questions: In your experience of accompanying your loved one through the transition from driver to license revocation, what did you find difficult? Since your loved one stopped driving, what changes have occurred in your life? The aim of these interviews was to gain a better understanding of the experience of family members accompanying older persons in their transition from driving to ceasing driving. At the end of the interviews with older persons and family members, sociodemographic data were collected.

### Data analysis

The sample size of the participant groups could be modified as empirical data saturation was reached when, as the interviews progressed, the answers became repetitive and redundant [33]. However, due to the small sample size, data saturation was not achieved.

All interviews were audio-recorded and fully transcribed in verbatim form immediately or couple days after the interviews. Using an inductive approach, the qualitative data obtained from the interviews was analyzed using the content analysis method [33]. Qualitative data were broken down into smaller units, then coded and named according to the content they represented. Finally, the coded material was grouped according to common concepts [33]. The first author analyzed the data, and then met the second and the fourth authors to discuss and agree on the list of themes to reach a consensus. NVivo software [37] was used to organize and code the themes and categories that emerged from the verbatim. In addition, descriptive statistical analyses were carried out on the socio-demographic data questionnaires gathered at the end of interviews with Microsoft Excel software. As this study was qualitative, Lincoln and Guba’s (1994) [33] scientific criteria (credibility, dependability, confirmability, transferability, authenticity) for qualitative studies were used.

## Results

### Participants

Among the elderly participants, five older persons had their driver’s license and were driving, two older persons still had their license but had stopped driving, and one older person no longer had a driver’s license (Table 1). Their average age was 78, and they had a high level of education. Finally, seven out of eight older persons were retired.

**Table 1:** Sociodemographic sample characteristics

Characteristics	Frequency	Mean	Range
Older persons (*n* = 8)			
Gender			
Female Male	5		
	3		
Age			
65 to 74 years old 75 to 84 years old 85 to 94 years old	4	78,37	67–91
	3		
	1		
Education			
High school diploma College diploma Bachelor’s degree or higher	1		
	2		
	5		
Civil status			
Single Widower Married/In couple	1		
	4		
	3		
Life situation			
Living with spouse Living alone	3		
	5		
Occupation			
Full-time job Part-time job Retired	0		
	1		
	7		
Frequency of car use (days/week)			
0 1 to 3 4 to 7	3		
	2		
	3		
**Caregivers (** ***n*** ** = 6)**			
Frequency of car use (days/week)			
Gender Female Male	4 2		
Age			
55 to 65 years old 66 to 75 years old	5 1	62	56–68
Education			
High school diploma College diploma Bachelor’s degree or higher	2 1 3		
Occupation			
Full-time job Part-time job Retired	2 1 3		
Relationship with older persons			
Sisterhood/Brotherhood Daughter/Son Other	1 4 1		
Possession of a driver’s license			
Yes No	6 0		

As for the relatives, the majority were under 65, and were the children of the older adults they accompanied. Lastly, half the relatives were still working, while the other half were retired. All relatives had a driver’s license.

### Qualitative results from interviews with older persons

Three main themes emerged from the analysis of the interviews: (1) The place of driving in older persons’ lives, (2) older persons’ perceptions of the driving assessment process, and (3) older persons’ lived or projected experience of driving cessation.

#### The place of driving in older persons’ lives

Older persons who still had their driver’s license reported using their car mainly to run errands, visit out-of-town family members, go to church or work. For these older persons, driving was a very important part of their lives, synonymous with freedom, independence, and a means of escape.

#### Older persons’ perception of the driving assessment process

The process of assessing fitness to drive was discussed by several older persons, most of whom were unfamiliar with it. In addition, most older persons had never discussed driving with a healthcare professional. Few older persons had discussed the subject of driving when they had their family doctor fill out a driver’s license renewal form. Half of the older persons said they didn’t think it was a good idea to discuss the subject with a healthcare professional, partly because they felt that they were fit to drive. For example, one older person said: “Personally, I wouldn’t see the point. Because at least, under the circumstances today, in the state I find myself in.” (A1). In addition, some older adults mentioned that they would accept to be assessed as to their ability to drive by any professional authorized and qualified to do so. Finally, a few older persons mentioned that they would like or would have liked to receive more information when they stopped driving, such as a list of resources, a list of alternative means of transportation or a list of community organizations. One older person who had stopped driving said he didn’t know where to turn for such information. He said, “Maybe I could have gotten some, but I didn’t really know where to turn.” (A2).

#### Older persons’ lived or projected experience of driving cessation

A few of the licensed older persons mentioned that they had never thought about the possibility of having to stop driving. Four older adults said they had no worries about the possible loss of their driver’s license. One said: “It’s because I’m still in good health that I have no fears as such. Maybe if I were sicker… And then I might have fears in the future, but since that’s not the case…” (A1).

Several factors were identified by older persons as positively or negatively influencing the experience of ceasing to drive. Firstly, the older people maintain that the place where they live influences cessation of driving. A few older persons mentioned that it was more difficult to stop driving when the place of residence was far from services, as evidenced by this excerpt: “I’m not close to amenities. I still need my driver’s license to go shopping.” (A5). One older person also pointed out that it was easier to stop driving when services made home deliveries, such as having his medication delivered by the pharmacy.

Transportation alternatives also emerged as a factor positively or negatively influencing the older persons’ experiences of ceasing to drive. Firstly, the accessibility of alternative means of transportation was raised by most older persons as a factor influencing driving cessation. Some older persons pointed out that finding alternative means of transportation represented a challenge. For others, access to alternative means of transportation was easier: “I traveled a lot by bus. […] I went pretty much wherever I wanted. Because a lot of buses pass by my house.” (A4).

The third factor to emerge from the interviews with older persons is the presence or absence of a network of friends and family which might facilitate transportation and mobility. Indeed, one older person pointed out that it can be more difficult to stop driving in the absence of a network of loved ones. He says: “For someone who doesn’t have family there… someone who doesn’t have a close family, it’s not easy.” (A5). Conversely, the presence of a support network to transport them or help them run errands was raised by three older persons.

Preparing to stop driving was also raised by older persons. One older adult who had stopped driving mentioned that it had been harder for him to stop driving, because he hadn’t had time to prepare for the transition. He says: “I wasn’t ready either. I wasn’t staying in an easy place to get my things, […] in my head I think I wasn’t ready.” (A2). On the other hand, some older persons mentioned preparation and planning to stop driving as a facilitating factor.

The fifth factor was the process of deciding to stop driving. One older person pointed out that it was more difficult to stop driving when the decision was imposed by the influence of people around him. One older person also pointed out that it was much easier to agree to stop driving when the decision was made by the older driver himself/herself.

The presence or absence of self-criticism is also a factor that can positively or negatively influence whether older persons stop driving. The presence of self-criticism enables the older person to make an accurate judgment of his or her ability to drive. For example, one older person says: “I thought my eyesight… I didn’t think my eyesight was very good either. It seemed to me that I couldn’t see as well as before, so that’s a feeling I had. […] Here and now, it’s safer for me and for others” (A4).

Finally, the older person’s understanding of the financial savings that come with ceasing to drive can facilitate this transition. For example, one older adult pointed out in interviews that he had saved several thousands of dollars by ceasing to drive, which reinforced his decision: “It’s economical! Cars are expensive! […] With insurance, it cost me $1,000. And parking in the residence was $1,000 per year. So right away, I saved $2,000 by leaving. Another incentive to stop.” (A4).

All older people discussed the impact of ceasing to drive on their lives. The older persons who had stopped driving discussed the actual impact, while the older persons who were still licensed discussed the anticipated impact of ceasing to drive on their lives. The five older person drivers still holding a license all said that driving cessation would have a major impact on their lives. Some older persons mentioned that it would bring about a lot of changes, particularly because their current place of residence was far from services and amenities, and they would be forced to move if they lost their driver’s license. Conversely, other older persons reported that stopping driving hadn’t significantly impacted their lives, mainly because of support from their loved ones. Older persons who still had their license also pointed out that stopping driving would have an impact on their loved ones, as they were the main drivers in the couple.

Several losses and bereavements were raised by older persons as being associated with ceasing to drive, such as the loss of independence. Indeed, they mentioned the difficulty of always having to depend on others to be able to get around. One older adult pointed out that he would have to mourn his role as the main driver if he had to stop driving. The loss of freedom and spontaneity was also raised by older persons.

### Qualitative results from interviews with caregivers

The analysis of interviews with caregivers will be presented according to two themes: (1) The elder’s driving cessation and (2) Family members’ experience of caring for the elderly.

#### The elders’ driving cessation

Several relatives mentioned feeling a sense of relief when older persons stopped driving:

“Looking back, you can’t wait until someone is injured or worse. Especially since there’s a daycare center right across the street from my parents’ house. It’s full of kids. I used to think “my God, what if he hurt someone”. So, it’s better this way.” (PR1).

Family and friends identified several factors that positively or negatively influenced the older persons’ decision to stop driving. These factors also had an influence on the relatives, as they accompanied the older persons in this transition and in their life without a driver’s license.

Three factors positively influencing the older person’s experience of stopping driving emerged from the interviews with relatives. Firstly, a relative maintained that stopping driving was easier when the older person agreed to take adapted transportation, and when he or she had the financial means to do so. Similarly, retirement from driving was easier, for both the older person and the relative, when the older person could get around independently in other ways. A relative also mentioned that it had been easier for the elder to stop driving, as he had obtained his driver’s license relatively late in life. As a result, the elder had previously been used to living without a license. A relative mentioned that it was easier to accept changes, such as the loss of a driver’s license, when decisions were made by the elder. As a result, the relative stressed that he tried to give the elder choices, so that the decision came from him. The relative said: “Decisions must come from them, you know the process… If it doesn’t come from them, all hell breaks loose. So, in the end, you plant the seeds of the idea, you limit the arguments, a little.” (PR4). Finally, a relative mentioned that it was easier for the elder to stop driving, since he had a network to help him get around and offer him support. The relative said: “We were able to meet his travel needs, whether it was for doctor’s appointments, going to the hospital, the ophthalmologist or whatever, we knew about his travel needs, and were all able to meet them.” (PR2).

On the other hand, three factors that had a negative influence on older persons’ experiences of driving cessation were highlighted by relatives. Firstly, one relative mentioned that it had been more difficult for the older person to stop driving, as he had not been prepared for it before: “Suddenly, we tell him, you can’t drive anymore.” (PR1). In addition, several relatives mentioned that it had been more difficult for older adults to stop driving, because they didn’t understand why they had to stop. Indeed, these older persons had an impaired capacity for self-assessment regarding their ability to drive, which made it more difficult for them to stop driving, according to their relatives. Indeed, the elders felt that they were still fit to drive safely and therefore did not understand the underlying reasons for ceasing to drive, as supported by a relative: “But she, my mother, she thought she was okay.” (PR5). In this sense, several relatives reported that older persons had attributed the reason for their failed road tests to people working in the legislative authorities or to their doctors. Finally, one relative mentioned that stopping driving was more difficult when the older person refused to have services to help him. In particular, the relative mentioned that the older person categorically refused volunteer transportation, because for the older person, “it’s like losing even more autonomy.” (PR3).

Indeed, some loved ones described the cessation of driving as a grieving process for the older adults they had accompanied. One family member mentioned that the older person felt he had lost his freedom: “For him, it was a question of freedom, and then he lost that freedom. It was one more loss.” (PR1). Two other relatives associated stopping driving with a loss of autonomy. One said, “Losing a driver’s license or giving up a driver’s license, well, it’s a bit like mourning because it’s like admitting… admitting that you’ve given up your autonomy.” (PR2).

#### Family members’ experience of caring for the elderly

During the interviews, relatives shared their experience of accompanying an older person who had stopped driving. Half of the relatives reported that they had not discussed the driving ability of the older persons they accompanied with a healthcare professional. Some reported having briefly discussed the older person’s ability to drive with a healthcare professional, such as a geriatrician, social worker, or general practitioner.

#### Impact on caregivers’ life

The older person’s cessation of driving had an impact, not only on the lives of the relatives we met, but also of other friends and loved ones. Several relatives mentioned that stopping the older person’s driving impacted their lives, because they had to transport the older person. One family member said, “It takes up a lot more of my time because I’m the one driving the taxi now.” (PR1). There was also an impact on another relative, the elder’s wife, as she became the couple’s main driver. The relative said:

“My mother still has her license, so now she’s driving for him. […] And my mother keeps it just because of my father, because she could have stopped driving, it would have suited her. Because she feels obligated to do the shopping and all that, and she doesn’t like to drive.” (PR1).

In contrast, other relatives mentioned that stopping driving had very little impact on their lives, because they were already used to and adapted to transporting the elder, or because the elder already had all the necessary services at home. A relative (PR6) mentioned that the elder’s cessation of driving had caused stress in his life, since he was afraid that the elder would “take his vehicle” even if he didn’t have a license.

Older persons’ driving cessation also had an impact on family dynamics. Firstly, a relative pointed out the change in roles that occurs following the cessation of driving: “He’s not used to being reprimanded or guided by his daughter. It’s quite the opposite. And now, it’s like… Again, it’s a stage where he doesn’t understand. I don’t have to give him advice. In his mind, he knows what he must do”. (PR1). Relatives also pointed out that differences had arisen between siblings in terms of their reactions and ways of dealing with the older sibling’s cessation of driving, which sometimes led to friction and tension within the family unit: “My two brothers were perhaps more adamant than I was. My older brother was like ‘Well, take his keys away’, because he continued to drive without a license for several months.” (PR1).

#### Challenges faced by loved ones

Disagreements were also experienced between relatives and elders, as they did not share the same perception of the elder’s ability to drive. While they reported that the older persons were unfit to drive, the elders disagreed. These differences of opinion caused conflict between the older persons and their relatives. One relative said, “He won’t see that he’s a bad driver, no matter how much I tell him.” (PR1). Several conflicts were also reported by relatives in connection with the fact that the older persons continued to drive without a license for several weeks or months following its revocation.

Analysis of the interviews with family and friends revealed several challenges associated with older persons’ cessation of driving. Firstly, many of the challenges raised by relatives were related to the fact that the older persons had cognitive deficits. Some loved ones mentioned memory deficits as a challenge, as the older persons didn’t remember losing their license. For example, one family member mentioned that it was difficult to enforce the recommendation not to drive. Similarly, some relatives mentioned that it was difficult to get older persons to understand the reasons behind ceasing to drive.

Secondly, some caregivers mentioned that they had misunderstandings associated with the process of assessing and terminating older persons’ driving, which presented a challenge for them in supporting their older persons.

Some relatives raised maintaining the bond of trust with the older person as a challenge. For example, one family member said: “That’s what’s hard. It’s this sort of, it’s tricky to keep… how can I put it? Not to lose the person’s trust. And at the same time, it gets tricky. You know it’s there, that you must move on to another stage, but…” (PR4).

Another challenge reported by loved ones was not knowing how to deal with older persons. They said they didn’t know how to deal with older persons who were angry about losing their driver’s license. As one family member put it, “We don’t have the words, we don’t have the way to do it, and we get resentment from our loved ones although we’re there to help them.” (PR6).

Selling the car of an older person who has stopped driving was also identified by some loved ones as a challenge. Some loved ones mentioned that they found it difficult to hear older persons talk incessantly about losing their driver’s license. For example, one loved one said, “Everyone would hear about it for months, it was kind of hellish that way.” (PR4).

#### Support received by caregivers

All the family members mentioned that they had not received any help or support from a person or organization in connection with the older persons’ cessation of driving. Several factors came into play, explaining why they had not received any help or support. Firstly, one relative mentioned that he didn’t know that he could get help from organizations. The relative said: “We never asked. We hadn’t either, we didn’t even know it existed.” (PR2). In addition, relatives mentioned that they did not tend to seek help, and sometimes refused to allow others to care for the elder. One relative also mentioned that he had asked for help but had not received any response to his request. He said: “There’s no one available. I asked, over there, at the center, to have a social worker […] who could call me to give me tips as they say, to know how to handle this, to know how to go with this.” (PR4). A family member mentioned that he had contacted several professionals for information and support, but that they were unable to answer him due to a lack of knowledge about the process of assessing fitness to drive. On the other hand, all the relatives mentioned that they would have liked to have received some kind of support. For example, one caregiver mentioned that he would have liked to know more about the steps and the assessment process with the legislative authorities. He would also have liked to know how to accompany the older person in his retirement from driving. One loved one also mentioned that he would have appreciated more information on the possibilities of support and alternative transportation, such as adapted transportation, if the loved one was unavailable to drive the older person.

## Discussion

The aim of this study was to describe the experience of older persons and caregivers in the transition from driving to driving-cessation. Firstly, analysis of the results showed that driving occupied a central place in the lives of older persons, and was synonymous with freedom and independence, as other studies have already reported [14, 15, 28].

Interestingly, most of the older persons and families we met were unfamiliar with the legislation and driving assessment process behind the maintenance of driver license. Older persons and family members had rarely discussed the subject of road safety with healthcare professionals. Similar information emerged in the literature, including the stress felt by relatives and older persons, due to their lack of knowledge about the licensing system, and a lack of awareness of how to obtain information and support [8, 10, 29]. Similarly, interviews with participants highlighted the anxiety and stress experienced by older persons and loved ones, throughout the process.

Older persons and caregivers reported that cessation of driving was associated with many losses and grief for older persons, such as the loss of independence and the role of driver, as well as the loss of freedom, spontaneity, and autonomy. Similar results have been reported in other studies [14–17, 30], where loss of freedom, independence, role, and occupation were also reported. The loss of a license is considered as one of the most difficult moments in older persons’ lives [28]. In fact, it seems that older adults who gave more importance to driving-related identities had more difficulty adapting to alternative solutions, such as the use of public transportation, than those whose identity was not linked to the accessibility and autonomy offered by driving [10]. Moreover, the literature highlights the challenge for older persons to maintain their activities and mobility, while depending on others to get around [15].

Analysis of data revealed seven factors (place of residence, transportation alternatives, network, preparation to driving cessation, decision-making process, self-criticism, financial savings) that positively or negatively influence the experience of ceasing to drive. Several studies in the literature review also put forward such factors, such as the older person’s ownership of the decision, the presence of a network of friends and family, and self-criticism [9, 10, 30, 31]. In doing so, experiential and empirical data demonstrate the importance of considering these factors, since they can be the focus of targeted interventions to ease cessation of driving. In fact, one study showed that an intervention facilitating the cessation of driving was useful and relevant for older persons, promoting their social participation and the resumption or continuation of significant activities [32].

Some of the older persons we met had never thought that they might one day lose their driver’s license. As a result, the older persons had not been able to prepare and modify their transportation habits prior to ceasing to drive, making the process more difficult for them. These situations have been linked to events where the elderly tended not to accept the recommendations of the legislative authorities and to go ahead and drive without a license [9]. The literature review and experiential data also highlighted the major impact that cessation of driving can have on the lives of older persons and their families [9, 30].

Cessation of driving can also have a significant impact on loved ones. For example, people who depend on older persons for transportation may experience significant loss if the older person must stop driving and may require psychological support. Loved ones who accompany older persons in the cessation of driving also face a number of challenges, such as assuming the entire burden of travel, psychologically supporting older persons in their grief/loss, and navigating the driver’s licensing system [8, 10, 29, 30]. Indeed, a phenomenological qualitative study carried out in the United States reports that relatives feel a sense of obligation towards the elder, who depends on them to get around. A feeling of burden, duty and additional responsibility is reported by relatives [30]. Some even feel uncomfortable or anxious about becoming the older person’s primary driver [10].

The data also highlighted the challenges faced by loved ones in supporting older persons who stop driving, particularly regarding memory deficits, where older persons couldn’t remember that they had lost their license. This challenge has also been raised in other studies [10, 31], stating that most active older drivers diagnosed with major neurocognitive disorders had difficulty understanding and accepting the results of their assessment, leading to almost inevitable debate and disagreement between the two parties. A relative recruited for one study even claimed that the announcement of the cessation of driving had a far greater impact on the older person than the announcement of the diagnosis of Alzheimer’s disease [31].

Cessation of driving by the elderly also had an impact on family dynamics, particularly in terms of role changes and how the situation was managed among family members. To this end, in the study by Ralston et al. [30], family members mentioned that stopping driving was accompanied by a restructuring of the family unit. Relatives who had taken on the role of child all their lives, now had to take on a new role as “parent” or caregiver. A second study [10] reports that some relatives described conflicts resulting from the elder’s difficulty in adapting to the new role, and attempting to maintain his identity as a driver by criticizing the conduct of family caregivers. In addition, more difficult relationships were raised at the level of the parent-child relationship, due to the authority dynamics that are present [10]. Finally, while some older persons feel comfortable asking their loved ones for support, others may be reluctant to depend on their family/friends to get around, as they are aware of the possible burden associated with this [16, 17].

Older people and relatives in this study mentioned that they would like or would have liked to receive more information when they stopped driving, such as a list of resources, of alternative means of transportation, or of community organizations. Comparable results are also found in the literature, arguing that loved ones need support and practical information to help them plan for the future of the older person without an automobile [8]. The same study revealed that older persons and their loved ones want both practical support to prevent isolation, and psychological support to help them through the transition process [8, 9].

There are several limitations to consider in this study. Interviews were conducted by telephone, which constitute a limitation since the nonverbal language of the participants could not be recorded. Moreover, conducting the interviews remotely was the only way to continue the study despite the COVID-19 pandemic. The small sample size may affect the external validity of quantitative data. In addition, the sample of recruited older persons had a high level of education, which affects the generalizability of the results. However, this study has some strengths. The qualitative design made it possible to have a more in-depth understanding of the experience lived by older adults and loved ones, and help targeting interventions opportunities for a better support of these clienteles and meet their real needs. The semi-structured interviews gave participants the freedom to express themselves and allows participants for open-ended responses for more in-depth information. The results of this study are transferable to another settings, which constitute a strength.

## Conclusions

This study provided insight on the transition of stopping driving from the perspective of older people and caregivers. Driving-cessation affects older persons and caregivers in various ways. This study highlights the distinct needs of older persons and caregivers related to the transition from driving to driving-cessation. Most of the older persons and families we met were unfamiliar with the legislation and driving assessment process, and older persons were not prepared to lose their driver’s license. Findings suggest that cessation of driving in later life is influenced by factors. It is interesting to know what these factors are, as it is possible to intervene in some of them to ease the transition to driving cessation. They also mentioned that they would have likes to have more information when they stopped driving (e.g., list of resources, list of alternative means of transportation), which shows that current support for older people and caregivers in the transition to ceasing to drive in Quebec, Canada, is inadequate. The results of this study will enable organizations and professionals to develop interventions that meet the real needs of older people and loved ones. With the aging of the population and increase in the number of older drivers, it is essential to take a more serious interest in this subject.

## Data Availability

The datasets generated during the current study are not publicly available, due to the risk of compromising individual privacy but are available from the corresponding author, upon reasonable request.
